# The ethical implications of verbal autopsy: responding to emotional and moral distress

**DOI:** 10.1186/s12910-021-00683-7

**Published:** 2021-09-04

**Authors:** Alex Hinga, Vicki Marsh, Amek Nyaguara, Marylene Wamukoya, Sassy Molyneux

**Affiliations:** 1 Kenya Medical Research Institute (KEMRI) - Wellcome Trust Research Programme, P.O. Box 230-80108, Kilifi, Kenya; 2 Nuffield Department of Medicine, Oxford University, Old Road Campus, Headington, Oxford, OX3 7LF UK; 3 African Population and Health Research Center, APHRC Campus, P.O. Box 10787-00100, Kitisuru, Nairobi, Kenya

**Keywords:** Moral distress, Verbal autopsy, Ethics, Emotional distress, Mortality data, Health demographic surveillance systems

## Abstract

**Background:**

Verbal autopsy is a pragmatic approach for generating cause-of-death data in contexts without well-functioning civil registration and vital statistics systems. It has primarily been conducted in health and demographic surveillance systems (HDSS) in Africa and Asia. Although significant resources have been invested to develop the technical aspects of verbal autopsy, ethical issues have received little attention. We explored the benefits and burdens of verbal autopsy in HDSS settings and identified potential strategies to respond to the ethical issues identified.

**Methods:**

This research was based on a case study approach centred on two contrasting HDSS in Kenya and followed the Mapping-Framing-Shaping Framework for empirical bioethics research. Data were collected through individual interviews, focus group discussions, document reviews and non-participant observations. 115 participants were involved, including 86 community members (HDSS residents and community representatives), and 29 research staff (HDSS managers, researchers, census field workers and verbal autopsy interviewers).

**Results:**

The use of verbal autopsy data for research and public health was described as the most common potential benefit of verbal autopsy in HDSS. Community members mentioned the potential uses of verbal autopsy data in addressing immediate public health problems for the local population while research staff emphasized the benefits of verbal autopsy to research and the wider public. The most prominent burden associated with the verbal autopsy was emotional distress for verbal autopsy interviewers and respondents. Moral events linked to the interview, such as being unsure of the right thing to do (moral uncertainty) or knowing the right thing to do and being constrained from acting (moral constraint), emerged as key causes of emotional distress for verbal autopsy interviewers.

**Conclusions:**

The collection of cause-of-death data through verbal autopsy in HDSS settings presents important ethical and emotional challenges for verbal autopsy interviewers and respondents. These challenges include emotional distress for respondents and moral distress for interviewers. This empirical ethics study provides detailed accounts of the distress caused by verbal autopsy and highlights ethical tensions between potential population benefits and risks to individuals. It includes recommendations for policy and practice to address emotional and moral distress in verbal autopsy.

**Supplementary Information:**

The online version contains supplementary material available at 10.1186/s12910-021-00683-7.

## Background

Despite the well-acknowledged benefits of reliable mortality data, about two-thirds of countries in Africa lack effective systems for generating data on number and causes of death [1]. A well-functioning civil registration and vital statistics system (CRVS) is often the most comprehensive source of reliable mortality data because it aims to register and certify all deaths in a country or region [2]. This universal registration and certification of deaths can benefit society and individuals, for example, by providing population-level mortality data to evaluate progress towards sustainable development targets or death certificates to facilitate inheritance processes [3].

The verbal autopsy is a pragmatic approach for generating data on causes of death in settings without a well-functioning CRVS, mostly implemented within health and demographic surveillance systems (HDSS) in Africa, Asia and Oceania [4]. Verbal autopsy involves interviewing a close relative or final caregiver of a recently deceased person to collect data about the signs, symptoms and circumstances that preceded death [5]. These data are analysed by physicians or automated computerized models to assign probable cause of death [6].

Global health stakeholders such as the World Health Organization and the International Network for the Demographic Evaluation of Populations and their Health (INDEPTH Network) have led international efforts to advance and scale up verbal autopsy methods [7–9]. They have developed and promoted electronic and automated approaches for collecting and analysing verbal autopsy data with the aim of reducing time, costs and data entry errors [10–12], and to enable wide scale use of verbal autopsy, including in CRVS [9, 13]. Verbal autopsy interviews are conducted by a wide range of stakeholders, including lay community members, health care workers and research field workers [14]. Overall, verbal autopsy has been implemented using diverse techniques to produce cause-of-death data primarily for research and surveillance, but also for CRVS.

Ethical issues in verbal autopsy have received little attention relative to technical aspects. The principal benefit of verbal autopsy is considered to be the provision of mortality data that can be used to support research and public health, including responding to disease outbreaks, such as COVID-19 [15]. However, many countries in low-resource settings have limited capacity for using data, such as global health estimates, and translating research evidence into public health benefits [16–18]. At the same time, researchers have described potential burdens of verbal autopsy for individuals and communities, including emotional distress for verbal autopsy interview respondents and stigmatization of bereaved families [19–21]. Some have argued that adhering to local bereavement practices, engaging community members throughout the verbal autopsy process and using data locally is crucial for minimizing burdens, enhancing benefits and showing respect in verbal autopsy [22–24]. Nevertheless, there has been little empirical research on the experiences of verbal autopsy stakeholders in HDSS within Africa.

We conducted an in-depth case study in two HDSS sites in Kenya to explore ethical issues in HDSS-linked verbal autopsies, increasingly focusing on benefits and burdens as the study progressed. In addition, we interviewed research staff working in HDSS outside Kenya to assess the wider relevance of the case study findings. In this article, we examine the nature and balance of benefits and burdens for a range of community and research stakeholders, including emotional and moral distress for verbal autopsy respondents and interviewers. While we did not set up this study to investigate moral distress, we found moral distress a useful conceptual framework for understanding the ethical, emotional and social challenges encountered by community and research stakeholders in verbal autopsy.

Andrew Jameton originally defined moral distress as “ *arising when one knows the right thing to do, but institutional constraints make it nearly impossible to pursue the right course of action*” [25]. Over time, scholars have either supported and applied this original definition [26] or recommended consideration of a wider range of causes and manifestations of moral distress [27–29]. We adopt the conceptualization of moral distress by Morley et al. 27, where moral distress is; (1) the experience of a moral event, such as an inability to do what one thinks is right (moral constraint), being unsure of the right course of action (moral uncertainty), moral tension, conflict or dilemma, (2) the experience of psychological distress, such as guilt and powerlessness, and (3) a direct causal relation between (1) and (2).

Most studies on moral distress have focused on social and health care workers, particularly nurses [27]. These studies have associated moral distress with negative impacts, including high staff turnover, burnout and compassion fatigue among health care workers [30, 31]. Consequently, institutions have implemented strategies to support staff, such as promoting greater interactions among different cadres of health care workers, with the aim of reducing or preventing moral distress [32–34]. However, some have argued that moral distress is not an entirely negative phenomenon; the experience of moral distress can reflect favorably on the character of an individual, provide opportunities for moral lessons and highlight systemic weaknesses [35, 36].

**Fig. 1:**
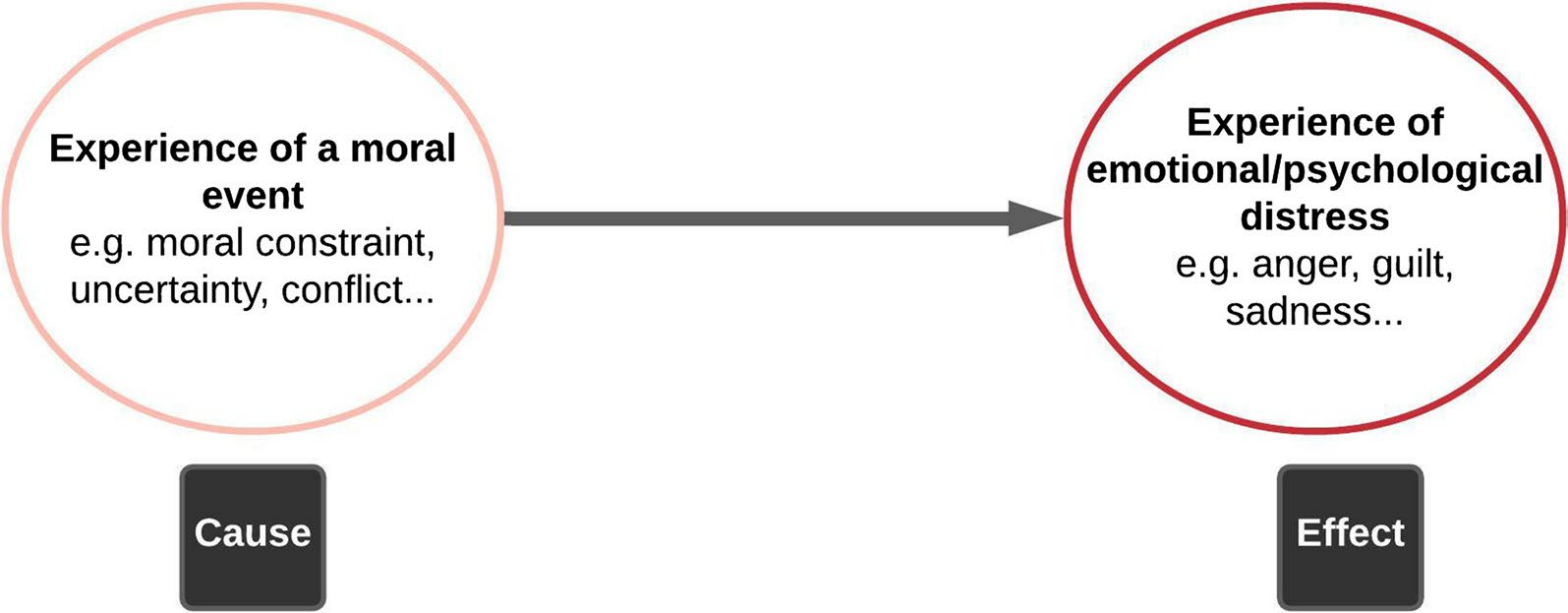
Moral distress conceptual framework

We conducted this study according to conventional standards for empirical bioethics [37–39] whereby we combined empirical data with the wider social science and ethics literature to explore the key ethical issues in verbal autopsy and to make recommendations on how research stakeholders should address these ethical issues. We applied the moral distress conceptual framework, which comprises the two components of moral event and emotional distress, as shown in Figure 1. The case study design enabled us to integrate the empirical and normative components of this study. We will describe the study design and methods, present our empirical findings around benefits and burdens of verbal autopsy, then discuss the implications of our findings for ethics policy and practice in verbal autopsy.

## Methods

### Study design

The case study reported in this paper was part of a larger (PhD) project on ethical issues in HDSS [40]. The larger project included three interlinked phases as outlined in the Mapping-Framing-Shaping framework for empirical bioethics research projects [37]. The Mapping phase involved literature review and formative research in diverse sites across sub-Saharan Africa to identify key ethical issues in HDSS [41]. It highlighted verbal autopsy as an ethically sensitive component of HDSS. This case study constitutes the Framing and Shaping phases of the larger project. It aimed to explore, in depth, ethical issues for verbal autopsy in two HDSS sites in Kenya (Framing) and to develop recommendations on the issues identified (Shaping). To assess the wider relevance of findings from Kenya to other sites across sub-Saharan Africa, we interviewed HDSS managers and researchers in Ghana, Malawi, South Africa and Uganda. We selected these sites based on scientific and pragmatic considerations including sites with diverse geographic, historical and organizational features (Table 1), and where the study team had social access.

**Table 1:** Key features of HDSS sites

Host country	Site urbanicity	Size of site (km^2^)	Population size (approx.)	HDSS inception Year
Ghana [42]	Rural	7162	140,000	2003
Ghana [43]	Rural	1675	156,735	1992
Kenya [44]	Rural	891	280,000	2000
Kenya [45, 46]	Urban	5.5	88,974	2002
Malawi [47]	Rural	135	39,000	2002
South Africa [48]	Rural	438	139,250	2000
Uganda [49]	Rural	28	23,000	1989

The philosophical underpinning of this case study was pragmatic constructivism [50, 51], where we acknowledged that reality is constructed through lived experience and interactions with others, and therefore used diverse research tools and multiple sources of evidence to explore different perspectives and co-create meaning. A “case” in this paper refers to a health and demographic surveillance system. The unit of analysis—benefits and burdens of verbal autopsy - emerged through progressive focusing [52], which involved mapping out ethical issues in HDSS across sub-Saharan Africa and exploration of ethical issues for verbal autopsy followed by in-depth investigation of benefits and burdens of verbal autopsy in two Kenyan cases.

### Study sites

The Kenyan cases were contrasting HDSS sites with language and cultural familiarity to the authors, facilitating rich data collection, analysis and interpretation. These included the Kilifi HDSS (KHDSS) and Nairobi Urban HDSS (NUHDSS) in Kenya. KHDSS is located on the coast of Kenya in the rural county of Kilifi. It was established in 2000 by the KEMRI Wellcome Trust Research Programme, a long-term multidisciplinary and quasi-governmental health research institution. Covering around 280,000 people, KHDSS is the largest INDEPTH Network site by population size [44, 53]. The NUHDSS operates in two urban slum areas (Korogocho and Viwandani) in Nairobi, Kenya’s capital city, where it follows up 88,974 people [45, 46]. It was started in 2002 by the African Population and Health Research Centre (APHRC), a non-governmental Africa-led institution [54]. Other sub-Saharan African sites involved in this study were rural based with diverse characteristics, but all operated by quasi-governmental and non-governmental health research institutions.

### Study participants and methods

In case study and non-case study sites, we purposively selected individuals with knowledge or experience of HDSS verbal autopsy practices and with a range of professional, social and demographic characteristics. We categorized participants into two main groups; community stakeholders (HDSS residents and community representatives), and research stakeholders (HDSS managers, researchers and routine census and verbal autopsy interviewers). This categorization was important for achieving a stratified purposive sample at the time of data collection. However, individual participants had responsibilities and identities that crossed the boundaries of the broad categories. For instance, some research stakeholders were residents in the HDSS areas and all verbal autopsy interviewers had previously worked as routine census interviewers. 115 individuals participated, some of whom had personal lived experience of conducting or participating in verbal autopsy interviews.

### Individual in-depth interviews across six HDSS sites

We interviewed 29 research staff involved in verbal autopsy policy and/or practice (Table 2). The individual in-depth interview is an appropriate tool for eliciting lived experience and enables the researcher and study participant to co-create meaning through conversation. Research staff in Kenya were interviewed in person while those elsewhere were interviewed by telephone or online. The guide for initial interviews covered HDSS ethics in general, including consent, community engagement, benefits, burdens and data sharing, with probes on ethical issues in collecting data on deaths, pregnancy status and household income (see Additional file 1). Detailed summaries immediately after interviews contributed to preliminary analyses and on-going revision of interview guides. Analysis of the first 11 interviews highlighted the ethical sensitivity of verbal autopsies in HDSS and led to the guides for the remaining interviews focusing on ethics in verbal autopsy; particularly benefits and burdens. All interviews were audio recorded.

**Table 2:** Individual interview respondents

Professional role/gender	Case_study_Site_1 (M/F)	Case_study_Site_2 (M/F)	Other_HDSS_Sites 3-6 (M/F)	Total
Researcher	4/4	0/2	1/1	12
Manager	4/0	2/1	2/1	10
Field Worker	2/1	2/2	0	7
Total	15	9	5	29

### Focus group discussions

We conducted 12 face-to-face focus group discussions with 86 HDSS community stakeholders (residents and community representatives) and field workers in the case study sites (Table 3). We chose the focus group method because it allows exploration of perspectives and experiences within a community. The total number of FGD participants was determined by the specific features of each site, membership of organized groups within the sites and availability of participants. The Nairobi Urban HDSS consists of two study areas located about 7km from each other. In each of the study areas, we held an FGD with one group of HDSS residents and another group of community representatives. The community representatives groups in NUHDSS, referred to as community advisory committees, had 10 members each. We invited all 20 members to participate in FGDs within their respective areas and 15 attended. In addition, we held an FGD with a group of NUHDSS census interviewers with experience of working in the two study areas. Overall, we facilitated five focus group discussions in NUHDSS involving 40 participants. Operationally, the Kilifi HDSS area is divided into three zones; northern, central and southern zone. We held an FGD with community representatives in each of the three zones and with HDSS residents in the northern and southern zones. In addition, we had separate FGDs for census and verbal autopsy interviewers. Overall, we facilitated seven FGDs in KHDSS involving 46 participants.

**Table 3:** Focus group participants

Focus group Discussion code	Participant group	Gender		Total	Duration (min)
		M	F		
FGD01_Site1_Location-D	Routine census interviewers	1	3	4	60
FGD02_Site1_Location-D	Verbal autopsy interviewers	4	0	4	87
FGD03_Site1_Location-A	Community representatives	4	4	8	119
FGD04_Site1_Location-B	Community representatives	5	3	8	97
FGD05_Site1_Location-C	Community representatives	4	3	7	125
FGD06_Site1_Location-A	HDSS residents	4	4	8	142
FGD07_Site1_Location-C	HDSS residents	4	3	7	126
FGD08_Site2_Location-A	HDSS residents	4	4	8	82
FGD09_Site2_Location-A	Community representatives	5	3	8	90
FGD10_Site2_Location-E	Routine census interviewers	2	6	8	84
FGD11_Site2_Location-B	Community representatives	4	3	7	77
FGD12_Site2_Location-B	HDSS residents	3	6	9	60
	Total	44	42	86	1149

The FGDs started with an open discussion to build mutual understanding of the research institutions (KWTRP in Kilifi and APHRC in Nairobi), health research, HDSS and the verbal autopsy (See Additional file 1). This was followed by discussion of ethically relevant ethical issues in verbal autopsy within HDSS with a focus of the views and experiences of participants. The lead author facilitated the discussions in English (1 group) and Kiswahili (11 groups). Discussions were audio recorded.

### Non-participant observations and document reviews

Seven verbal autopsy interviews were observed in Site 1 (n=5) and Site 2 (n=2). The number of interviews observed was determined by practical considerations, including availability of interviewers to accompany to the field, and data saturation. In Site 1, one interviewer was observed during a full working day, where they conducted five interviews. Since the first two observations in Site 2 did not contribute new themes, there were no further observations. The interviews involved diverse respondents in terms of age, gender and relations to the deceased, with the deaths described linked to accidents, suspected suicide, childbirth and chronic illness. Verbal autopsy questionnaires, consent forms, benefit and data sharing guidelines, and the INDEPTH Network website were reviewed for information on methodological and ethical processes for verbal autopsy in HDSS.

### Data management and analysis

We managed the data using NVivo 10, Microsoft Word and Excel. Participants were not identified by name in the audio recordings and field notes. Also, details that could risk identification of participants, such as specific roles of participants, are excluded in this article. All data were stored in a password protected computer, while devices and hard copy documents were stored in a locked cabinet.

We analysed the data following the Framework Approach [55, 56]; this involved transcription, familiarization, development of a thematic framework, indexing, charting, mapping and interpretation. Transcripts from audio recordings of interviews and focus group discussions, field notes from observations and summaries of document reviews were typed into Microsoft Word and Excel. Eleven FGD transcripts were translated from Kiswahili to English by a senior field worker at the KWTRP who has extensive experience in translating health research documents. The lead author listened to all the audio recordings and read the transcripts and field notes to familiarize with the qualitative data across the study. Three authors (AH,SM,VM) independently coded three interview transcripts and developed an initial thematic framework influenced by predominant themes in the ethics literature, such as consenting, burdens and benefits, and by issues emerging from the data. This framework was applied to the rest of the data and charts developed, using NVivo 10 Framework Matrices, to highlight patterns across sites, themes and participant groups. The overall data were interpretated through consultations within the study team and against the wider ethics and social science literature. The key empirical evidence was around burdens, specifically emotional distress for interviewers and respondents, while moral distress was used as a conceptual framework for understanding the ethical and social determinants of emotional distress.

## Results

We describe the views and experiences of study participants around the benefits and burdens of verbal autopsy in HDSS, focusing on emotional distress as a core emerging issue. The focus for this study was on the Kenyan sites, where we conducted in-depth research, accompanied by exploratory research outside Kenya. The study results reported below reflect the varying depths of engagement in case study and non-case study sites. The term “VA stakeholders” refers to community and research stakeholders as defined earlier. We report study results under three main themes; (a) benefits and burdens for all VA stakeholders, (b) emotional distress for VA respondents, and (c) emotional distress for VA interviewers.

### Overview of benefits and burdens in verbal autopsy

#### Benefits for VA stakeholders

Different study participant groups emphasized different potential benefits of verbal autopsy. Community stakeholders felt that verbal autopsy data could be used to control local public health and social problems, including hereditary diseases, infectious disease outbreaks and violence.

“…there are those inherited diseases…it will reach a point to ask ‘was there another person… great-grandfathers who died because of this disease? That can help [the research centre] know that these diseases are in this family…” Community-Representative_Site-1

“I would like this organization to find how they can help us… and some of our children who are being killed by gunshots… to get out of this environment.” Community-Member_Site-2

In contrast, most research staff emphasized that the benefits of verbal autopsy are likely to emerge after a long time and for the broader public, because the findings are generally used for research and reported as aggregate mortality data. The few research staff who did mention potential benefits of verbal autopsy at the individual and local community level, focused on relief from emotional distress for respondents,

“We have found that people find it [VA interview] quite cathartic. When people have been distressed by the death, they find it rewarding that somebody is interested, and somebody cares about how and why that person died.” HDSS-Manager_Site-3

Some verbal autopsy interviewers described gaining knowledge and experiencing satisfaction in helping community members by talking through their loss. Every working day, interviewers listen to respondents describing a wide range of illness and circumstances that lead to death, and some felt this had helped them to be more aware of health risks and how to manage these.

“I have learned so much, like neglecting [minor illnesses] can lead to death. I did a VA interview for someone who had died from meningitis. They had tonsilitis, the tonsils burst, so the infection spread to the brain and they got meningitis. I have learned so much, even that I should not ignore a child who has high temperature.” VA-Interviewer_Site-2

Learning about the cause of death of a family member or friend was highlighted as a potential benefit of verbal autopsy because it could help the bereaved get closure or take measures to reduce the risks of morbidity and mortality.

“I know from experience of groups who have done minimally invasive tissue sampling … that families sometimes might be put at rest by understanding what caused death. I think that might also apply to the verbal autopsy if the family don't know what caused the death...” Researcher_Site-1

“…after researchers know that in a certain family, the mother had a certain sickness and it has manifested again in the child, maybe they could follow up on that family … they could test the other children who are alive to know who could be affected.” Community_Representative_Site-1

However, other participants pointed out that informing the bereaved family about the cause of death might not always be helpful. This is mainly because the accuracy of verbal autopsy varies according to context and the cause of death might be obvious, for example, death from road traffic accident.

“I think one of the problems with verbal autopsy is that it is relatively imprecise… , so it’s not that much of a comfort to the family, and I’m sure a lot of family members will say “so what’s the answer?”, well, I could have figured that out myself.” Researcher_Site-1

### Burdens for VA stakeholders

Discussions with participants and field observations underlined that the verbal autopsy can include burdens for all stakeholders. One example was the risk of stigmatization and discrimination of communities if aggregated causes of death, especially deaths associated with stigmatized diseases and crime, are reported by region, sex, age or ethnicity.

“If it is known there is a lot of HIV in [this area], people will avoid this area fearing infection, even doctors might refuse to come and work here”. Community-Member_Site- 2

Gaps in community understanding of verbal autopsy procedures and objectives were also seen as potentially generating suspicions, rumours and a sense of injustice, leading to institutional reputational damage and impacting behaviour.

“We [research centre] are often perceived as being part of the hospital if not the hospital itself. If we annoy people, they may be unwilling to use health care services…” Researcher_Site-1

Other potential burdens included time costs and invasion of privacy for verbal autopsy respondents and bereaved families, and risks to the personal safety of interviewers, especially when seeking to collect data on deaths in contexts of family conflicts, suspected witchcraft and tensions between bereaved families and government authorities. For example, local chiefs may prohibit funeral gatherings for various reasons, which could make the bereaved resent visitors associated with authorities.

“… In a funeral [where there have been conflicts between the bereaved and local authorities], a researcher cannot go there to ask the cause of death, you may go there and they [take] revenge on you.” Community-Representative_Site-2

### Emotional distress for verbal autopsy respondents

Perhaps unsurprisingly, emotional distress emerged as the most common and serious burden for verbal autopsy respondents. Study participants recognised that the death of a relative or friend, and discussions of that death, are likely to cause or exacerbate emotional distress, referring to emotions such as grief, guilt, helplessness, frustration, anger and anxiety.

“…when you go there to talk about a deceased person, maybe that person [respondent] is like ‘we have gone through this, we have talked about it’ and you are reminding him or her.” VA-Interviewer_Site-2

“…the primary issue is that it’s just a really painful thing to go and talk, as a stranger, to somebody who has experienced losing somebody who is close to them…” Researcher_Site-1

Since the verbal autopsy interview entails asking respondents to recount the circumstances of death, it may introduce or worsen feelings of guilt, helplessness and self-blame.

“…not everybody wants to revisit the problems that led to a death, maybe there is some self-blame, children died suddenly, and they feel they didn’t do enough [to prevent it].” Researcher_Site-1

Other negative emotions for respondents might include annoyance, frustration and anger. These emotions were described in relation to perceived unfairness; where participants felt that the burdens were disproportionate to benefits for verbal autopsy respondents, as this comment from a resident who participated in a verbal autopsy interview after losing a spouse indicates:

“…when you have lost a relative, then someone comes here and starts to ask questions… you start feeling bad. So, you are hurting me and not helping me with anything, you can tell the person “go away! You are disturbing me”. Community-Member_Site-2

Also, despite attention to data confidentiality in HDSS guidelines and in practice, some respondents may be anxious that information from the verbal autopsy interview, especially for violence-related deaths, will be passed on to the police or other stakeholders, with negative repercussions for the respondents.

### Influences on emotional distress for verbal autopsy respondents

The main contextual influences on emotional distress for verbal autopsy respondents included cause of death, characteristics of the deceased, the relationship between respondent and deceased and the timing of interview.

#### Cause of death

Injuries and HIV/AIDS accounted for 16% and 32% of deaths in KHDSS and NUHDSS respectively. Deaths associated with violence, accidents and witchcraft were described as particularly sensitive for the bereaved to deal with and to report. Where death was related to a shooting (177 out of 5495 verbal autopsy interviews conducted in NUHDSS), potential feelings of anger, annoyance or frustration were often linked to a sense of injustice related to the cause of death, compounded by a lack of clarity on what the verbal autopsy interview sought to achieve in this context.

“When the police were trying to disperse protestors… a baby was shot dead accidentally near the road… if you go to do an interview with the parent they may fail to understand your research because they are angry that their innocent child is dead.” Community-Representative_Site-2

Since deaths from shootings were strongly associated with crime, respondents might feel anxious about the implications of providing cause of death information, or feel they could have prevented a death by supporting the deceased to change behaviour, generating a sense of guilt that verbal autopsy interviewers might exacerbate through their questions.

“Maybe this boy was killed by police…so, when the researcher asks, “Why was he killed, did you sit down and talk to him?” You know, that means I did not sit down and talk with that child and that is why he was killed…” Community-Member_Site-2

It would be unusual for an interviewer to ask the respondent whether they advised the deceased to avoid crime. However, the above quote suggests respondents might interpret some open-ended interview questions more broadly and find them judgmental.

Some HDSS community stakeholders felt that beliefs in witchcraft as a cause of death and illness are prevalent in the HDSS areas and that families with such beliefs may find the verbal autopsy interview frustrating, unnecessary or suspicious.

“When someone dies in this community, most times, bewitching must be mentioned [as the cause of death]. They brought my sister’s kid to me, I took him to the laboratory and he was tested for malaria, he did not have malaria. I took him for HIV testing, he was found to be [HIV] positive and was taken for counselling… later he became very weak and was admitted. His [other relatives] came and removed him from the hospital ward and took him to be treated with clean water, he died two days later, they say he died from bewitching.” Community-Representative_Site-1

Besides deaths from shooting and suspected witchcraft, participants felt that deaths associated with acute illness, homicide, road and work accidents and suicides, would be particularly sensitive for VA respondents.

“When someone hangs themselves, [respondents] might not be open. They might be afraid it will be known that they were not in good terms with the deceased… So, fear is a challenge” Community-Representative_Site-1

#### Characteristics of the deceased and respondent

The age of the deceased, their role in the family and the relationship between the main respondent and the deceased were felt to influence the sensitivity of verbal autopsy. Deaths, and associated verbal autopsies, were seen as less sensitive at extremes of age (neonates and the very elderly) compared to those of older children and other adults. For example, deaths of the very elderly might be viewed as natural and acceptable ends to suffering.

“For an old person who is so old until they are playing with their feces, if they die, you say this one should go and rest, but for a child you will feel pain.” Community-Member_Site-2

Greater sensitivity was seen for verbal autopsies following the death of family members with important socioeconomic roles, including household heads, family ‘breadwinners’ and community leaders and where the person interviewed was a close relative or friend of the deceased, particularly for a parent. Most participants acknowledged that experience of emotional distress was highly subjective.

“…like the saying goes ‘the mother knows the pain of childbirth’, so it depends on who you’re talking to about whose death.” Community-Representative_Site-2

#### Timing of VA interview

The timing of verbal autopsy interviews varied across sites and was described as an important influence on emotional distress for respondents. In the NUHDSS, the timing of verbal autopsy interviews depends on specific circumstance of death, while interviews in the KHDSS are generally planned three or more weeks following a death. In practice, most interviews (73%) in the KHDSS were conducted three or more months after death and within three months after death (58%) in the NUHDSS.

The most appropriate timing for interviews was difficult to establish. Conducting an interview ‘too soon’ was seen as potentially interfering with grief and offending mourning norms, contributing to greater emotional distress for respondents. At the same time, it was recognized that there may never be an appropriate time, with interviews conducted after a longer period still causing distress.

“… every time [that we tried to interview the respondent] she would break down and cry.” VA-Interviewer_Site-1

“If you go in earlier [to conduct verbal autopsy], you get more accurate data, if you delay you get more inaccurate data but equally if you go in early and you offend some social norms you won’t get any data at all.” Researcher_Site-1

### Emotional distress for interviewers

Verbal autopsy interviewers described their own emotional distress in collecting information around deaths, including sadness, guilt, regret, helplessness, powerlessness and anxiety. Most of these feelings were directly related to witnessing and feeling responsible for respondents’ distress, but also from noticing the health and socioeconomic challenges of bereaved families.

Interviewers described being saddened by accounts of suffering and being unable to forget disturbing details.

“In a day, you may get two or three cases, which you cannot forget.” VA-Interviewer_Site-1

“There was this boy who was shot… I interviewed his sister…she said…’yes; my brother was stealing, he had just been shot in the chest, but he was still alive, he told the cop[police officer], please forgive me’, the cop shot him in the head… sometimes I tear up while doing the interview.” VA-Interviewer_Site-2

Some interviewers may blame themselves or regret making respondents emotionally distressed, especially during interviews where respondents cry. Whenever interviewers encounter severe emotional distress, or health and socioeconomic challenges, they might be unable to help, contributing to feelings of guilt and helplessness.

“We are working in a community where we encounter so many challenges daily. You go into [a household] and find somebody without food, somebody who is sick, somebody who has been bereaved, somebody who has been raped and you don’t have any solution, because sometimes you are even broke.” VA-Interviewer_Site-2

Besides financial constraints to fully addressing the challenges of respondents, many interviewers felt they had inadequate counselling skills to comfort the bereaved.

“Trainings on counselling would help, instead of someone breaking down and you are left telling them sorry, sorry, sorry and it ends there” VA-Interviewer_Site-1

“…and not that in-house training, but training in a college… that’s where we can build our skills for helping the [interview respondent].” VA-Interviewer_Site-1

Formal capacity-strengthening initiatives had been undertaken at the institutional level, including intermittent bereavement counselling and communication skills training. Several HDSS managers and researchers felt that verbal autopsy interviewers would be able to address the challenges of collecting cause of death data, based on their training, experience and social capital, and had doubts about the kind of ‘training’ that would best support interviewers:

“[The verbal autopsy interviewer] is well known and liked in the community… I think people respond very well to him.” HDSS-Manager_Site-3

“… there is limited time for the homestead visit. It’s just unrealistic to expect someone to do a lot of good in terms of counselling. And I think that badly done counselling is probably more harmful than no counseling.” Researcher_Site-1

From our field observations, verbal autopsy interviewers collected data in a professional and respectful manner, seeming to draw on shared faith and cultural traditions to comfort the bereaved, as fellow community members.

At the same time*,* institutional policies can contribute to interviewers’ feelings of helplessness, powerlessness and frustration. Most HDSS sites operate with little formal integration with other health care and population information systems making it difficult for interviewers to refer respondents to professional health and social care services.

As a policy, two of the seven sites involved in this study gave ‘condolence fees’ to bereaved families following a verbal autopsy, as a token of sympathy. Even in these two sites, funding and other institutional restrictions at times prevented interviewers from giving condolence fees at the time of the verbal autopsy interview. Discussions with participants highlighted the difficulty in establishing an appropriate condolence fee that would signal appropriate respect from relatively wealthy research institutions, fit with local mourning norms and not raise expectations around research payments in general or personal contributions from interviewers.

Across all sites, most verbal autopsy interviewers saw condolence fees as a way of respecting local mourning norms, including showing solidarity with bereaved families, rather than reimbursing or influencing respondents.

“… when people are mourning, you mourn with them. So, I have come here to mourn with you, it’s not that I am buying you, I am mourning with you.” VA-Interviewer_Site-2

“It becomes very difficult for us, sometimes I may even go deeper into my pocket to contribute beyond what is given by the research centre when I see that these people [bereaved] are really stranded…” VA-Interviewer_Site-2

It seems likely that expectations from community members around condolence fees, coupled with personal and institutional constraints to issuing these, could create tensions for interviewers visiting bereaved households without the capacity to support. In general, interviewers’ reflections on their roles in collecting data and sharing information, and the balance of benefits and burdens for verbal autopsy respondents, seem to generate feelings of powerlessness and act in tension with personal values:

“So sometimes I feel like as much as I try to convince them [bereaved] that there is direct benefit or indirect benefit [of verbal autopsy], sometimes I feel like I’m lying, because the person has died and if there’s still an illness in the household, no one will come in to help them.” VA-Interviewer_Site-2

## Discussion

Previous studies have highlighted the potential burdens of verbal autopsy, particularly for interview respondents [14, 19–24]. Our study contributes additional empirical data and extends analysis of ethical issues for verbal autopsy in HDSS sites within sub-Saharan Africa, including burdens for diverse stakeholders. We further propose that most of the emotional distress for verbal autopsy interviewers constitutes moral distress. Taken together, burdens of verbal autopsy, including moral distress, generate important ethical responsibilities for those who implement or promote verbal autopsy, as we discuss next. We summarize our key recommendations for verbal autopsy in Table 4.

**Table 4:** Key recommendations for verbal autopsy practice

Issue	Recommendations	Justifications
Rationale for verbal autopsy processes	1. Collect verbal autopsy data from a wider range of respondents and sources, including community informants, health care workers, hospital and police records	It is ethical to use alternative methods of data collection when these methods minimize risks and provide data of similar quality
Condolences and compensation	2. Compensate VA respondents for time spent in interview and issue bereaved with a culturally appropriate token of condolence	To demonstrate respect for local community bereavement practices
Data sharing and use	3. Use verbal autopsy data locally	
	4. Integrate VA with CRVS to facilitate issuance of death certificates and strengthen mortality statistics	To ensure a fair balance of benefits and burdens among VA stakeholders
Interviewer recruitment	5. Consider diversity, including in terms of gender, age and religion, field experience and cultural competency when recruiting interviewers	Conducting VA interview presents significant emotional and social challenges. Interviewers draw on experience, cultural knowledge and bonding social capital to address these challenges
Interviewer training	6. Provide training on bereavement counselling	Counselling skills will enable interviewer to comfort distressed respondents
Interviewer support	7. Ethics reflection sessions and implementation of agreed action points 8. Establish a moral distress consultation service	Address interviewers’ emotional distress and strengthen ethics practice through group discussions and one-on-one conversations with ethicists and mental health experts

### Responding to emotional distress and other burdens of verbal autopsy

As shown in this article, verbal autopsy in HDSS within sub-Saharan Africa involves risks of a wide range of burdens for individuals, communities, institutions and health systems. Verbal autopsy respondents and interviewers are likely to bear the most common and severe burdens. Notably, the emotional distress of respondents is at the centre of most verbal autopsy related burdens.

Institutions that undertake health-related activities have an ethical responsibility to identify, monitor and minimise burdens; this might involve identifying populations that are susceptible to harm or injustice, modifying procedures or stopping an activity [57]. In this way, research institutions, HDSS networks, global health agencies, governments and individual researchers have an ethical responsibility to minimise emotional distress and other burdens of verbal autopsy and to enhance social value of the activity. In what follows, we outline potential strategies for addressing burdens at different levels and stages of verbal autopsy.

### Reviewing rationale and processes for verbal autopsy

A potential strategy for meeting the ethical responsibility for addressing burdens would be to review the objectives and processes of verbal autopsy processes in each site. For example, while the INDEPTH Network requires verbal autopsy procedures for all deaths in affiliated HDSS sites, some of these data may be available from other sources. In this study, cause-of-death data related to shooting, road traffic accidents or suicide are likely to be available from community leaders, community health volunteers and medical records from health facilities affiliated to HDSS. Collecting such data from alternative verbal autopsy respondents seems particularly important since many of these sudden and traumatic causes of death generate high risks of emotional distress. Emotional burdens for interviewees could also be reduced by expanding the pool of potential respondents for verbal autopsies to avoid involving particularly vulnerable individuals. Studies have shown that home-based birth attendants and health care workers can substitute for bereaved mothers as verbal autopsy respondents and provide credible information about stillbirths and neonatal deaths [58]. Given the risks involved, a call for verbal autopsy for all deaths in HDSS, which often involves interviews with close relatives of the deceased, needs reassessment in each site.

### Condolences

International ethics guidelines for research involving humans recommend that participants should be compensated for inconvenience and time spent in research [57]. Compensation and payments to research participants often generate significant ethics debate and uncertainty with concerns around undue influence, coercion and exploitation of participants [59]. Policies in most sites involved in this study did not include payments or compensation for time for verbal autopsy respondents. Some may object to compensation or payments for respondents citing risks to individual autonomy, such as undue influence or coercion, and the introduction of unsustainable financial expenses for research institutions. They may also argue that verbal autopsy is not research or that it involves minimal inconveniences and time costs for respondents.

However, protecting individual autonomy is not the only or most important concern for health research in LMICs; addressing social injustices, and providing benefits and minimising burdens are equally important concerns [60]. In addition, as a form of epidemiological research, HDSS verbal autopsy is closer to a research than a public health activity and can involve substantial inconvenience and time costs for respondents. More emphasis should therefore be placed on compensating respondents for inconvenience and time spent in a verbal autopsy interview. Also, providing a condolence fee or token to the bereaved family would have the added benefit of demonstrating respect for cultural norms.

### Ensuring fair balance of benefits and burdens

Given the sometimes important burdens generated for VA respondents, it is important that these are adequately balanced by forms of benefit, to promote ethical practice [20]. Verbal autopsy contributes to global health estimates, but these estimates might not be useful at the local level [16]. Furthermore, public health policies based on global health estimates that exclude HDSS verbal autopsy data might not be significantly different from policies drawn using estimates that include these data [61]. The additional value of verbal autopsy data to global health estimates and the benefits of these estimates to countries that contribute the data is unclear. Nevertheless, most of the standardization of verbal autopsy processes seeks to generate data that are comparable across sites and countries. Also, the barriers of translating research evidence into policy and practice are well acknowledged [16, 62]. Based on the empirical data in this study on burdens of verbal autopsy, we join others in recommending integration of VA with CRVS [9, 13, 63, 64] and engagement of community members throughout the verbal autopsy process [22, 65], to minimize burdens and enhance benefits. Besides supporting research and contributing to global health estimates, it seems that there should be greater emphasis on the importance of verbal autopsy data being used to benefit individuals, local communities and health systems directly, including by facilitating issuance of death certificates and informing responses to local public health problems.

In cases where verbal autopsy gives reasonably reliable information on cause of death at the individual level, we argue that research stakeholders have an ethical obligation to share this information with individual respondents and bereaved families. Also, population-level data from verbal autopsy should be shared and used for public health benefit with acknowledgement of data limitations to demonstrate transparency and respect. For example, some have proposed a classification of mortality data based on quality of cause-of-death assignement [66], which could be useful in informing appropriate uses of verbal autopsy data. The wider sharing of verbal autopsy data could enhance benefits for individuals and local communities and the efficient use of resources. Given the risks of community stigmatisation, emotional distress for individuals and supplanting national health information systems, the sharing of verbal autopsy results should be accompanied by engagement of all relevant stakeholders, including research field workers, health workers, the bereaved and government.

### Moral distress for verbal autopsy interviewers

From the data, we consider that most of the emotional distress among verbal autopsy interviewers is best understood as moral distress. Moral distress has been described as the experience of psychological (emotional) distress in relation to the experience of a moral event [27] (as illustrated in Fig. 1). When compared to other HDSS stakeholders, verbal autopsy interviewers seem most vulnerable to moral distress. While HDSS managers and researchers described experiencing moral uncertainties and dilemmas concerning verbal autopsy, there were no indications that they experienced emotional distress in response to ‘moral events’. Also, most of the emotional distress for verbal autopsy respondents, such as grief, was not related to experience of moral events. In contrast, the emotional distress of verbal autopsy interviewers, such as helplessness, frustration and guilt, was often linked to moral uncertainties, conflicts, dilemmas and constraints to actions in keeping with their moral values, such as increasing grief for bereaved families without the capacity to respond in culturally appropriate ways.

Constraints to moral actions can contribute to moral distress, including external influences such as institutional policies that prevent individuals from acting in accordance with their moral values [25, 27]. For example, most interviewers felt that they should issue culturally appropriate tokens to bereaved families and refer respondents for professional health and social care but could not because of restrictive institutional policies. Constraints to actions can also be internal or personal [67]. Verbal autopsy interviewers described a perceived lack of skills in bereavement counselling and lack of adequate personal resources to comfort or alleviate the suffering of respondents and bereaved families.

Besides constraining moral actions, institutions can constrain the ability of individuals to act as autonomous moral agents based on professional hierarchies [68]. Verbal autopsy interviewers are among the most experienced field workers, often having supervision and management responsibilities. However, the ‘field worker’ role is generally considered a junior position; this group have lower academic qualifications and training compared to research staff [69, 70]. Interviewers may play little to no role in decision-making for HDSS, but they are required to implement and explain HDSS policies to respondents and fellow field workers, even if they disagree with some decisions. In nursing contexts, this has been framed as treating individuals as ‘mouth pieces’ for others, a potential cause of moral distress [68]. The actual or perceived lack of power, skills or knowledge to act otherwise, given institutional policies, can lead to moral distress for verbal autopsy interviewers.

Moral distress can occur even when individuals act as they wish [27, 29]. Interviewers described making out-of-pocket financial contributions to demonstrate solidarity with the bereaved, advising respondents and adapting consent procedures to context. These actions may have a negative impact on interviewers’ personal finances and careers where they do not align with institutional guidelines and policies. Taking action to protect one’s values, despite personal and professional risks, has been recognised as a distinguishing feature of moral distress [35].

In addition to barriers to moral actions and constraints to moral agency, individuals can experience moral distress in relation to moral uncertainty, dilemma, conflicts or tensions [27, 29]. The verbal autopsy in HDSS presents a wide range of practical and ethical challenges. These include uncertainty around the appropriate timing of verbal autopsy interviews, the necessity and impact of reporting findings to individual respondents and the appropriate ethics guidelines for verbal autopsy in HDSS contexts. Also, the verbal autopsy generates ethical dilemmas for interviewers in situations where their personal values and responsibilities are in conflict or tension with local cultural practices or institutional ethics policies [22]. Interviewers acknowledged the potential social value of verbal autopsy data and at the same time felt that they were contributing to burdens for respondents by collecting the data. Some questioned the social value of verbal autopsy. These challenges may contribute to moral distress for verbal autopsy interviewers in HDSS.

### Verbal autopsy interviewers: recruitment, training and support

We join others in recommending more investment in institutional support and capacity development for research field workers, including recognition of the physical, social and emotional challenges they may encounter, and including context-specific training and provision of career opportunities based on institutional recognition of field workers’ complex roles [69, 71]. Training in ethics, bereavement counseling and project management with certification could support interviewers to fulfill the responsibilities of their complex roles and to advance their careers. In addition, the regular revision of verbal autopsy tools should be accompanied by consideration of the impact of these revisions on interviewers and respondents. For example, a brief interview, despite reducing time costs, might appear unsympathetic to the bereaved, hence contributing to emotional and moral distress.

While pragmatic considerations (such as ability to ride a motorbike), and assessment of cultural competency (such as ability to speak local language) in recruitment often mean that most verbal autopsy interviewers are male and from large local ethnic groups, a deliberate decision to recruit interviewers with diverse personal characteristics in terms of gender, age, ethnicity and religion could promote ethical practice since these interviewers would have a high likelihood of leveraging on bonding social capital [72], to minimise burdens for themselves and respondents. Finally, interviewers need ongoing support to address emotional and moral distress. This could include establishing permanent platforms that enable interviewers to discuss ethical challenges they encounter, and to recommend changes to institutional policies and practices. While we recommend further research to assess the impact of moral distress on verbal autopsy interviewers’ health and careers and acknowledge that moral distress is an unpleasant experience for individuals, we also recognize the potential value of moral distress [36], including in identifying and addressing ethical issues in verbal autopsy. Interventions such as regular ethics reflection sessions [73, 74] and moral distress consultation [34] for verbal autopsy interviewers can contribute to addressing emotional distress and strengthening ethical practice.

## Conclusion

Verbal autopsy in HDSS may provide potentially valuable data on causes of death at population level but may involve substantial costs for HDSS residents and verbal autopsy interviewers, which we argue could be avoided or reduced. The population benefits of verbal autopsy need further empirical research in diverse settings. We have highlighted that verbal autopsy respondents and other HDSS residents experience significant uncompensated burdens and that verbal autopsy interviewers experience forms of emotional and moral distress.

Notably, verbal autopsy respondents seem to bear the most severe burdens while gaining the least benefits. Scholars have investigated ethical issues for verbal autopsy in research and routine settings [9, 14, 19–24, 75, 76]. To our knowledge, this is the first study to apply the moral distress framework in understanding the emotional, ethical and social challenges in verbal autopsy practice. We contribute to the moral distress literature, which predominantly draws from research in health care settings, and provide specific recommendations for VA stakeholders.

The uncompensated burdens for HDSS residents, including emotional distress for respondents, and moral distress for interviewers generate important ethical responsibilities for those who promote or implement verbal autopsy. The key responsibilities include responding to emotional and moral distress and ensuring a fair balance of benefits and burdens. We have suggested that HDSS research stakeholders should review the need for verbal autopsy in each site to reduce unnecessary burdens for interview respondents and bereaved families. In addition, we propose that verbal autopsy respondents in HDSS should be compensated, including condolence fees, not only to reduce burdens for stakeholders but also to demonstrate respect and solidarity. Finally, there is a need for institutional platforms and policies to respond to interviewers’ moral distress and to promote the greater use of verbal autopsy data at the local community level.

## Supplementary Information

**Additional file 1**. Interview and focus group guides: HDSS ethics study.

## Data Availability

The datasets used and analysed during the current study are available from the corresponding author on reasonable request.

## Abbreviations

AAS: African Academy of Sciences
AESA: Alliance for Accelerating Excellence in Science in Africa
APHRC: African Population and Health Research Centre
CIOMS: Council for International Organizations of Medical Sciences
CRVS: Civil Registration and Vital Statistics Systems
COVID-19: Coronavirus Disease 2019
DELTAS: Developing Excellence in Leadership, Training and Science
HDSS: Health and Demographic Surveillance System
IDeAL: Initiative to Develop African Research Leaders
IDI: Individual in-depth interview
INDEPTH: International Network of field sites with continuous Demographic Evaluation of Populations and Their Health
KEMRI: Kenya Medical Research Institute
KHDSS: Kilifi HDSS
LMICs: Low- and middle-income countries
NEPAD: New Partnership for Africa's Development Planning and Coordinating Agency
NUHDSS: Nairobi Urban HDSS
REC: Research Ethics Committee
SERU: Scientific and Ethics Review Unit
VA: Verbal autopsy
WHO: World Health Organization
