# Supplementary material for: The ethical implications of verbal autopsy: responding to emotional and moral distress
Source: BMC Med Ethics. 2021 Sep 4;22:118. doi: 10.1186/s12910-021-00683-7 (PMC8418286; doi:10.1186/s12910-021-00683-7)
Supplement: Supplementary file 1 — Additional file 1. Interview and focus group guides: HDSS ethics study. [file 12910_2021_683_MOESM1_ESM.pdf]

1. HDSS Ethics Study: Sample Individual Interview Guide – March 2017

Interviewee identifier:

Date & place of interview: ____

Time start and end: _____________ to _____________

Interviewed by: ________ Note taker: ________

Voice recorded? [Y] / [N]

General comments on interview:

VIEWS EXPERIENCES

1. What are your duties and responsibilities within this HDSS?
2. In your usual duties and responsibilities, which aspects of the Health and Demographic Surveillance Systems do you mostly interact with*? Community-based interventions/Clinical Surveillance/HDSS management/secondary data analysis/ scientific and ethics review…*
3. What do you consider to be the core features/main platform of this HDSS? [*Monitoring of vital events, malaria/clinical surveillance?]*
4. What do you think are the main strengths of a HDSS? Strength of *HDSS as a methodology, justification for HDSS, effectiveness, acceptability…*
5. What do you think are the risks and benefits of HDSS to the people involved in its operations? *Researchers, community members, health facilities, research institutions…*
6. In your work within the HDSS, have you ever encountered an issue that you would consider ethically challenging? [*Informed consenting, community engagement, data sharing, benefit sharing, accountability, governance?]*
   - *Can you give me some examples?*
   - *Which are the most important/common ones and why?*
   - *What are the formal/informal systems in place here to address these challenges?*
   - *How well do these systems work?*
7. Do you think consent should be obtained for collection of HDSS data? If yes, from whom and why (*individuals, household heads, community leaders, government…*) when (*at establishment of HDSS, at each visit, annually, new households, in-migrants…*) How (*written, verbal?).*

8. Have you been involved in disseminating the findings of HDSS to community members? How (*e.g community meetings, radio*) and when (*before/after publication*) was this done?
9. Do you think HDSS proposals and tools should undergo scientific and ethics review? If yes, why and how often (*annually, each re-enumeration round, new tools only…*) which REC (*CSC,SERU, both?*)
10. From whom should permission (if any) to re-use HDSS data be obtained? [*Individuals, community residents, researchers, institutional/national review committees*]
11. Should community members be involved in HDSS data collection e.g CKI? If yes/no, why?
12. How can community members and community interests be represented in HDSS agenda setting, review and data governance?
13. Are you aware of any harms or benefits that have emerged from the collection, use and sharing of HDSS data? *Can you give some examples?*
14. What strategies – community engagement, research agenda setting- do you think would help (or have been effective) in understanding and responding to HDSS residents' concerns and needs?

2. HDSS Ethics Study: Sample individual interview guide – November 2018

Roles within Research Centre/HDSS Site

15. How long have you worked with "Research centre /HDSS site"? *What are your current roles and responsibilities? Have you had different roles/positions in the past? Experience working with HDSS site?*

16. **<u>Views and Experiences of Ethical Issues</u>**

We all face challenges of different kinds in our work. Some challenges are quite practical (e.g having the right kind of computer or vehicles) and some are more ethical. While working with X have you faced any ethical issues or dilemmas? i.e any issues or situations that made you uncertain/wonder/reflect about what the right thing to do is? *Can you give some examples? Which are the most important ones? When did this happen?*

[If nothing forthcoming] - For example some people have told us about dilemmas or being unsure about issues related to the amount or type of information that people in communities are given, or about what to do when they encounter people with health needs.

[Verbal Autopsy is a key component of many HDSS sites, it has emerged as an important area of focus in my study. I would like to ask you a few questions on specific ethical issues for VA].

Verbal Autopsy

- The Verbal Autopsy is an indirect technique for determining the cause of death.
- It involves interviewing close relatives, friends or caregivers of a recently deceased person to find out the signs, symptoms or circumstances that preceded death.
- This information is then analysed by medical doctors (Physician Certified VA) or by software applications (e.g Inter-VA) to assign the probable cause of death.
- VA data is important because it provides public health authorities with evidence to inform policies, planning and implementation of public health interventions.
- The majority of HDSS sites (47/53) conduct Verbal Autopsies on deaths that occur within their HDSS area.

[I value the insights you'll be able to give on the HDSS and VA in particular.]

Benefits of VA

1. I would like us to talk about the value of VA. What do you think are the benefits of VA to stakeholders? *Can you give some examples?*

   - *VA respondents/bereaved families – emotional support, learning about causes of death, condolence fees…*
   - *HDSS Residents – Support to health care system, public health interventions…*
   - *HDSS researchers/field workers – data to support projects, career development…*
   - *Ministry of Health/research centres – Informing policy, interventions, programme evaluation*
   - *Global Community – Advancing science, knowledge of global burden of disease*

Burdens of VA

2. Could you mention any specific problems that you think the VA might generate for the people and institutions involved?

   - *VA respondents/bereaved families*
   - *VA interviewers*
   - *Research centres*
   - *Community stigmatisation*
   - *Supplanting the local health systems – e.g CRVS, competition for staff or opportunity costs e.g doctors taking time to assign CoD…*
3. In your opinion, what influences the problems associated with the VA interview? *Type of death, timing of interview, characteristics of deceased/respondent/interviewer…*

Strategies to Maximize Benefits and Minimize Burdens

4. In X HDSS site, VA respondents/bereaved families are given a condolence fee of about $4 after the interview. *What are your views on this practice? Do you think issuance of condolence fee would be necessary/feasible/acceptable in your HDSS site? Why…*
5. Some HDSS stakeholders have suggested that VA interviewers should receive training on bereavement counselling so that they can offer emotional support to VA respondents. *What are your thoughts on this suggestion? Can you share any ideas on how VA benefits can be maximized and burdens reduced?*

6. When should the VA interview be conducted and for what reasons? *After burial, X days/weeks/months after death…*

Weighing Benefits and Burdens

7. Would you say that benefits and burdens associated with VA are fairly distributed among different groups of HDSS stakeholders? *Prioritizing public health benefits over individual burdens, sense of unfairness to respondents, field staff women…* why/why not?

8. Overall, what do you feel about the balance between benefits and burdens for VA? Is it worth doing at all?

Consent and Community Engagement

9. What do you think HDSS community members understand about the Verbal Autopsy? *Who conducts VA, how and why?*
    a. What type of information should they have and for what reason?
    b. *Is there anything more that you think could be done to increase community understanding of VA? What more and for what reason?*

10. How does the HDSS gain knowledge about local bereavement practices e.g mourning period? How is this knowledge used to design and implement VA?

    a. *Community engagement activities for VA?*
    b. *Is there anything more you think could be done to increase research stakeholders understanding of death and bereavement practices in the local community? What more and for what reasons?*

11. *I have learnt that some HDSS sites obtain individual written consent from respondents for collection of Verbal Autopsy data while others obtain verbal consent.*
    a. *How is consent obtained for VA in your HDSS site?*
    b. *In your opinion, how should consent be obtained and for what reasons? Verbal/written, from whom, when…*

Data Sharing and Use

12. Who has access to the VA data collected in your HDSS site? *Who can access, when, how and for what purposes…*
    What are your thoughts about the current VA data sharing arrangements?
    - *In terms of fairness, efficiency, accessibility, protecting privacy and interests of participants?*
    - *How well are community members interests represented when sharing VA data?*

13. What criteria do you think should be used in considering who can have access to the VA data?
    - *Type of institutions? Where based, the reason data is being sought, existing partnerships, type of data requested?*

14. Can you give any examples on how VA data from your HDSS has been used? e.*g research projects, policies, PH interventions, advocacy…*

Oversight and Ethics Review

15. Have the VA protocols and tools been reviewed by a scientific and ethics committee? *Why not/ if yes, how often* (*annually, new tools only…*) which committees?
    - *Should VA be subjected to an ethics review process? For what reasons?*

16. Who should monitor VA implementation and be involved in decisions on ethics review and governance? *How and for what reasons?*

    - *HDSS tool standardization and audit vs review by ethics committee*

3. HDSS Ethics Study: Sample Focus Group Discussion Guide – November 2017

FGD code [__][__][__][__]   Date ____________________ Place ____________________

Throughout - explore what participants understand about KEMRI, KHDSS and Verbal Autopsy. Ask questions first before sharing information and then asking more.

A – Understanding of KEMRI and health research

- ***Does anyone know what KEMRI is and what it does?***
    - *Government institution (State Corporation) responsible for carrying out health research in Kenya.*
    - *Works in partnership with MoH, whose responsibilities include making health policies, responding to public health concerns such as outbreaks and delivering health care services in Kenya.*
- ***Does everyone understand what health research is?***
    - *Health research involves systematically collecting and analysing information or samples in order to increase our knowledge about a particular health issues.*

B - Explore participants' understanding & experiences of KHDSS

- ***Have any of you had any experience of the KEMRI census activity? Do you know much about what information they are collecting and why?***
- ***What of that information is collected regularly, and less regularly? What information is more and less sensitive?***
- The census includes about 280,000 people in 16 locations - since 2001.

- KEMRI field workers visit homesteads every 3-4 months to collect information about individuals - e.g who has moved in or out - households e,g source of drinking water – and about buildings – e,g type of building material and location of building.
- The fieldworkers obtain verbal consent before asking questions. Any knowledgeable member of the household who is ≥13yrs can give consent and provide information on behalf of other household members.
- The collected information is stored safely in password protected computers.
- KEMRI works together with the Ministry of Health; this enables information collected from homesteads to be linked with that collected from the Kilifi County Hospital. This information is analysed by KEMRI researchers. It may also be shared with other researchers outside KEMRI.
- The census has enabled KEMRI to determine the number of people living within the KHDSS area, their ages, migration patterns, diseases among other important details about people's lives.

**Frequency**	**Less Sensitive**	**More Sensitive**
**Every time**	Births, migration, deaths, residence status, building units, building location (GPS data)…	Pregnancy**, Verbal Autopsy**
**Sometime**	Ownership and use of bed nets, source of drinking water, sanitation facilities, vaccination status, education level…	Income, ownership of economic assets, National ID…

C - Explore participants' understanding & experiences of Verbal Autopsy

- The death of a family member or a friend is usually a difficult thing for many people to bear. If a person living within the KHDSS dies, KEMRI fieldworkers visit the homestead to ask further questions about the death. What do you know about when, where, how and why these questions are asked?
- What are some of the important things for KEMRI to learn about local community practices around death, burials and mourning? How might KEMRI learn about these things?
- As far as you are aware, are there any differences between the Verbal Autopsy interview and the routine census interview? *In terms of how permission to go ahead is obtained, who conducts the discussions, duration of interview, setting…*

Verbal Autopsy

In settings with well-functioning health and vital events registration systems, the majority of deaths are officially recorded and the cause of death given. In addition, a death certificate is issued. The cause of death is determined based on information collected in health facilities and the report of a pathologist. Enumeration and certification of deaths is important because it enables public health practitioners and policy makers to understand the causes of death in a population and to inform interventions.

In many African countries, the majority of people receive care away formal health care facilities and die at home. Furthermore, when an individual dies, post-mortem investigations are rarely conducted. This makes it difficult to determine the number of deaths and their causes. The lack of this information creates challenges for efforts aimed at reducing morbidity and mortality.

The Verbal Autopsy is commonly used in areas without effective death enumeration and certification systems such as Kilifi.

- The Verbal Autopsy is an indirect technique for determining the cause of death.
- It involves interviewing close relatives, friends or caregivers of a recently deceased person to find out the signs, symptoms or circumstances that preceded death.
- Unlike the census field workers who visit households every 3-4 months, VA field workers visit a household only when a death has been reported.
- Usually, VA interviews are conducted by experienced field workers with basic counselling skills.
- In KHDSS VA interviews are conducted at least 21 days after a death has occurred.
- Information from VA interview is used to assign a probable cause of death.
- VA is not very accurate at determining the cause of death for an individual. However, when conducted for many people over a long period, VA gives researchers a good idea on causes of death in a community.
- Unlike in some research projects, VA respondents are not compensated for time they spend to participate in the interview. The VA interview can take about 30 minutes and is normally held in the participants' home.
- In the absence of reliable data from health facilities and government agencies on the causes of death, VA is the best available source of information on deaths.
- Researchers at KEMRI and others all over the world can use VA information to support other research projects while the government and global community can use VA to make policies and implement various health and development programmes.

Benefits and Burdens

1. What do you think about the approach that I've just described that KEMRI takes? Is there anything else that should be considered?
   - ***For example are there deaths that are particularly sensitive and require special handling?***
   - *Age, gender, religion and other personal details of the person who died*
   - *Relationship of the VA respondent to the deceased.*
   - *Treatment-seeking before death (from linked hospital data) and general circumstance of death?*
   - *Duration of interview*
   - ***What kind of changes in practice would you suggest are needed in different cases?***

2. What are some of the challenges that might emerge during and after the Verbal Autopsy interview?
    - *Emotional distress/anxiety/discomfort of the respondent and VA interviewer*
    - *Loss of income due to time taken for the VA interview*
    - *Breach of confidentiality*
    - *Stigmatization of the community based on causes of death reported*
    - ***How should these challenges be addressed?***

3. Recognising that these interviews will always be difficult is there anything that individuals and families might gain from participating in a Verbal Autopsy interview?
    - *A chance to share/discuss what happened?*

4. Has anyone here ever given a suggestion about how the Verbal Autopsy should be conducted (including a complaint)? How did this happen, how was it handled, and what did they feel about the outcome? [Collect views on any episodes from the group].

<u>Consent</u>

Before a VA interview in KHDSS, individual participants are asked to sign a piece of paper to show that a field worker has clearly explained Verbal Autopsy to them and that they have made a free decision to participate. The participant needs to be someone who was closely involved in caring for the deceased or someone with information about circumstances that led to a death. This is unlike the KHDSS census where verbal consent is obtained from any knowledgeable member of the household.

1. *What do you think about this consenting process for VA in KHDSS?*
    - *There are some HDSS sites where field workers still make sure that people have been informed and that they have made their own decision, but field workers do not ask people to sign a piece of paper about this.*
    - *What type of consenting do you think is most appropriate for VA interviews in KHDSS and why?*

<u>Community Engagement</u>

2. What do you think community members understand about the Verbal Autopsy? – why it's done and its' value.

3. KEMRI carries out various community engagement activities to create mutual understanding with KHDSS residents. These activities have included community meetings, open days at KEMRI, information leaflets and radio programme. They provide opportunities for community members to give feedback and ask questions about KEMRI. Before a Verbal Autopsy interview, field workers inform respondents about KEMRI, KHDSS and VA.
   - *What is the information that should be provided to the general community about VA and why do you think this information is important?*
4. At the moment, a separate team of KEMRI staff who are specially trained, come out to do VA interviews. *What do you think about this approach? Might there be a better one?*
   - *e.g health workers vs fieldworkers*
   - *At KHDSS it's only KEMRI census field workers who collect information about deaths in the community and share it with the VA interviewers. However, in some HDSS sites, in addition to census field workers, selected community members are trained to record deaths that occur in their neighbourhoods and to share this information with the HDSS at least once a month. VA interviewers then visit the household to collect further information. Would you support such an arrangement for KHDSS? What are the advantages and disadvantages of current KHDSS system of reporting deaths and conducting VA? Pros and cons of alternatives?*
5. Currently, the KHDSS does not report findings of VA to individuals and families that participate in the VA interview. This is because the Verbal Autopsy is not very good at determining the cause of death for one individual. However, the causes of death at the community level are regularly shared, including through publications in scientific journals.
      - *What do you think about this practice of reporting findings of VA in the KHDSS?*

   Should the findings of Verbal Autopsy be reported back to individual participants/families? Why and how should this be done?

<u>Data Sharing</u>

6. Data collected by KEMRI is stored safely in protected computers and cabinets. These data can be shared with others in and outside KEMRI to help them generate more knowledge, make policies or implement programmes. KEMRI has a committee that

reviews data requests to ensure that shared data is used for the right reasons. Who should have access to the verbal autopsy data that KHDSS collects and why?

- *KEMRI researchers, global research community, NGOs…*
- *Kilifi County and national government…*
- *Everyone with the intention of using data to improve public health…*

7. How should the interests and rights of VA respondents and KHDSS community be protected when sharing VA data?
    - *Community representatives in data governance committees…*
    - *Community consultation before sharing of VA data to others outside KEMRI…*
    - *Data anonymization – note difficulties of fully anonymising population-level Verbal Autopsy data.*
8. Would anyone here be interested in accessing VA findings/results? *If yes, VA findings for whom (family members, friends, neighbourhood, entire KHDSS) when (on request, monthly, annually…) for what reasons?*
9. Should VA data be treated differently compared to rest of KHDSS in terms of storage, analysis and sharing? Why/why not?

Oversight of the KHDSS

10. Who should permit and monitor the activities of the KHDSS including Verbal Autopsy? How and why?
    - *Ministry of Health, county government, KEMRI internal/national RECs, INDEPTH Network...*
11. Any further recommendations on how ethical challenges involved in Verbal Autopsy should be responded to?
12. Is the Verbal Autopsy worth doing at all? *Why/why not?*
